# Functional, biological, and radiological evaluation of the pancreaticojejunal anastomosis 1 year after pancreatoduodenectomy: a prospective study

**DOI:** 10.1007/s00423-023-03040-x

**Published:** 2023-08-22

**Authors:** Gaëtan-Romain Joliat, Pierre Allemann, Ismail Labgaa, Nicolas Demartines, Naik Vietti Violi, Sabine Schmidt, Markus Schäfer

**Affiliations:** 1 Department of Visceral Surgery, Lausanne University Hospital CHUV, University of Lausanne (UNIL), Rue du Bugnon 46, 1011 Lausanne, Switzerland; 2 Graduate School of Health Sciences, University of Bern, Bern, Switzerland; 3 Department of Diagnostic and Interventional Radiology, Lausanne University Hospital CHUV, Lausanne, Switzerland

**Keywords:** Pancreatectomy, Cancer, Permeability, Complication

## Abstract

**Purpose:**

This prospective study aimed to analyze the functional, biological, and radiological aspects of the pancreatic anastomosis 1 year after pancreatoduodenectomy (PD).

**Methods:**

From 2016 to 2019, patients with PD indication were screened. Questionnaires about pancreas insufficiency, fecal elastase tests, and magnetic resonance imaging (MRI) were performed before and 1 year after PD.

**Results:**

Twenty patients were prospectively included. The only difference between pre- and postoperative questionnaires was constipation (less frequent 1 year after PD). Median pre- and postoperative fecal elastase levels were 96 μg/g (IQR 15–196, normal value > 200) and 15 μg/g (IQR 15–26, *p* = 0.042). There were no significant differences in terms of main pancreatic duct (MPD) size (4, IQR 3–5 *vs*. 4 mm, IQR 3–5, *p* = 0.892), border regularity, stenosis, visibility, image improvement, and secondary pancreatic duct dilation before and after secretin injection. All patients but one (2 refused and 2 were lost to follow-up, 15/16, 94%) had a patent pancreaticojejunal anastomosis on 1-year MRI.

**Conclusion:**

Although median 1-year fecal elastase was significantly lower than preoperatively, suggesting that exocrine secretion was altered, the anatomical outcome as assessed by MRI was excellent showing high patency rate (15/16, 94%) at 1 year. This emphasizes the difference between anatomy and function.

**Supplementary Information:**

The online version contains supplementary material available at 10.1007/s00423-023-03040-x.

## Introduction

Pancreatoduodenectomy (PD) is performed for various etiologies, but mainly for pancreatic head cancers. PD is a complex and technically demanding operation, including pancreaticoenteric anastomosis. The pancreatic reconstruction is generally performed either with pancreaticogastrostomy or pancreaticojejunostomy. Each anastomosis has various techniques with specific advantages and disadvantages. Pancreaticogastrostomy is reported to have more postoperative hemorrhages, while pancreatojejunostomy more pancreatic fistulas [1, 2]. The short-term complications of pancreatic anastomosis, such as dehiscence, pancreatic fistula, or organ/space surgical-site infection, are frequent and well described after PD (30–40%) [3, 4]. Several predictive factors for these complications such as intraoperative main pancreatic duct (MPD) size or pancreas texture (soft/hard) have been identified [5–7]. A small (<3 mm) MPD size and a soft pancreas have been shown to increase the risk of postoperative pancreatic fistulas [5, 6].

Conversely, very few data exist on the long-term evolution of pancreatic anastomosis after PD. It is also unclear whether stenosis of this anastomosis can induce postoperative exocrine pancreatic insufficiency. Computed tomography (CT) allows the morphological assessment of MPD and pancreatic parenchyma. However, owing to the functional imaging capabilities and the excellent contrast resolution, magnetic resonance imaging (MRI) is considered superior to CT for the evaluation of postoperative pancreas, i.e., pancreatic anastomosis [8]. Furthermore, some articles suggested to use MRI with intravenous secretin injection in order to enhance visibility of the pancreatic anastomosis [8–10].

The aim of the present study was to prospectively assess the functional, biological, and radiological aspects of pancreaticojejunal anastomosis 1 year after PD.

## Material and methods

### Design and surgery

Patients who accepted the study were prospectively enrolled after informed consent. Inclusion criteria were adult patients (>18 years old) with PD indication for malignant etiologies. Chronic pancreatitis (due to potential preoperative pancreatic insufficiency), language barrier, contraindication to MRI (claustrophobia, metallic implant), or refusal to participate were all exclusion criteria. Patients with pancreaticogastrostomy were withdrawn from the study. The study recruitment extended from July 1, 2016, to July 31, 2019 (screening, data collection, and follow-up until July 2020).

Classic Whipple procedure with hepaticojejunostomy, pancreaticojejunostomy, and antecolic gastrojejunostomy (omega reconstruction) was performed in all patients. Pancreaticojejunostomy was routinely performed at our center, except for patients with very small MPD and soft pancreas in whom pancreaticogastrostomy was carried out. Postoperative complications were graded according to the Clavien classification [11]. Major complications were defined as grade III or higher. For each patient, the comprehensive complication index (CCI) was calculated [12]. Delayed gastric emptying, pancreatic fistula, and hemorrhage were defined according to the international study group for pancreatic surgery (ISGPS) [13–15]. Surgical-site infection was defined according to the Centers for Disease Control and Prevention [16]. All patients received pancreatic enzyme replacement (Creon) after PD for the entire study period (50,000 UI three times a day).

### Questionnaires

Preoperatively and 1 year after the operation, a questionnaire inquiring about clinical symptoms of exocrine pancreatic insufficiency was given to the included patients. The following items were part of the questionnaire: abdominal discomfort, bloating, heaviness feeling after meals, abdominal pain, greasy stools (steatorrhea), soft stools, smelly stools, diarrhea, urgency, constipation, gas, weight loss, eating with appetite and pleasure, nausea, and weight loss. Items were graded using a 5-point Likert scale (1 = always, 2 = most of the time, 3 = sometimes, 4 = rarely, 5 = never). Subjective feelings for the last 6 months were assessed. The questionnaire was evaluated and tested by two gastroenterologists from two different departments prior to the distribution. The questionnaire is available as Supplementary Material 1.

### Fecal elastase tests

Fecal elastase tests measure the concentration of pancreatic elastase-1 in the feces using an enzyme-linked immunosorbent assay. These tests were realized in a centralized laboratory. Stool samples were collected in hospital or patients collected a stool sample at home and sent them to the laboratory directly. Results were expressed in μg/g. The norm was >200 μg/g, defining the threshold for exocrine pancreatic insufficiency [17]. A fecal elastase level <15 μg/g was considered as severe exocrine pancreatic insufficiency. Tests were realized preoperatively between the preoperative consultation and the day before operation and postoperatively 1 year after the operation.

### Magnetic resonance imaging

**Fig. 1:**
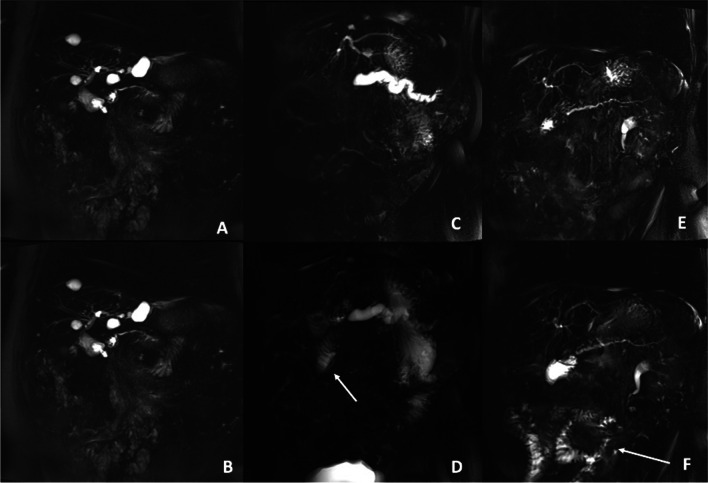
Dynamic pancreatography obtained at 0 (**A**, **C**, and **E**) and 10 min (**B**, **D**, and **F**) after intravenous secretin injection of three different patients, at 1 year after pancreatoduodenectomy. The first patient (**A**, **B**) evidenced no intraluminal secretion at 10 min after stimulation, suggesting reduced jejunal filling capacity. The second patient (**C**, **D**) evidenced filling between first and second anastomotic jejunal loop at 10 min (**D**, arrow). The third patient (**E**, **F**) evidenced filling of the first two anastomotic jejunal loops and more loops (**F**, arrow)

A preoperative MRI with gadolinium injection was realized maximum 1 month prior to PD. One year after surgery, all patients underwent conventional pancreas MR examination at 3 Tesla (MAGNETOM Skyra, Siemens Healthineers, Erlangen, Germany) using an 18-channel body array coil and a 32-channel spine coil during which dynamic MR pancreatography (MRP) was performed. Patients had been fasting for at least 5 h prior to MR examination. MRP consisted of oblique coronal breath-hold two-dimensional single-shot turbo spin-echo T2-weighted sequences acquired before and every 30 s for 10 min after intravenous (i.v.) injection of Secrelux® (secretin, 2 UI/kg, Sanochemia, Austria). The following MR features based on previous studies [8, 18] were monitored: baseline and maximal size, as well as the regularity of MPD (in mm), visualization of MPD side branches and presence of ductal narrowing, permeability of pancreatic anastomosis, jejunal filling progressively occurring after secretin injection, pancreas atrophy, and overall image quality. The latter was graded semi-quantitatively as no, slight, or major image improvement after i.v. secretin injection according to Monill et al. [8]*.* Jejunal filling was semi-quantitatively evaluated at 10 min and was graded according to the method of Matos et al. [18] as follows: grade 1 (no secretion or filling limited to the anastomotic jejunal loop), grade 2 (filling between first and second anastomotic jejunal loops), and grade 3 (filling of the first two anastomotic jejunal loops or more). Figure 1 illustrates this classification. Images were analyzed independently by two abdominal radiologists (NVV, SS) with 10 and 20 years of experience, respectively, who were blinded to all clinical information including patients’ outcomes.

### Pathology

Tumors were classified based on the 8th TNM edition of the American Joint Committee for Cancer.

### Statistics

Continuous data were compared using the Mann–Whitney *U* test and categorical data using the chi-square test. Patient survival was calculated using Kaplan–Meier method. Median follow-up was calculated using the reverse Kaplan–Meier method. Correlations were calculated using Spearman’s coefficients. Interobserver concordance (reliability) between the image analysis performed by the two radiologists was calculated using the weighted kappa coefficient of Cohen with 95% confidence interval (CI), and the ratings proposed by Fleiss were used (*k* < 0.4: poor agreement; 0.4–0.75: good agreement; >0.75: excellent agreement). All *p*-values were two-sided. A *p*-value < 5% was considered significant. All statistical analyses were performed using SPSS 26 for Mac OS X.

## Results

A total of 75 PD patients were screened, 53 were excluded and 22 accepted the study and were included. Two patients were withdrawn from the study because a distal pancreatectomy with splenectomy was performed instead of PD in one case and one patient had a pancreaticogastrostomy instead of a pancreaticojejunostomy, yielding a cohort of 20 patients (11 women, median age 66 years, interquartile range, IQR 58–74) for final analysis. Details are shown in the flow chart (Supplementary Figure 1). Patients’ characteristics are depicted in Table 1. Surgical, pathological, and postoperative details are shown in Table 2. Etiologies for PD were ductal adenocarcinomas (*n* = 13), cholangiocarcinomas (*n* = 3), ampullary carcinomas (*n* = 2), and duodenal carcinomas (*n* = 2). Seventeen patients had postoperative chemotherapy. Among the 13 patients with ductal adenocarcinoma, all of them received adjuvant chemotherapy (gemcitabine-based regimens: *n* = 9, FOLFIRINOX: *n* = 4). Mean overall survival of the entire cohort was 40 months (95% CI 32–48). Twelve patients developed a tumor recurrence during a median follow-up of 30 months (95% CI 25–35).

**Table 1:** Characteristics of the included patients (*n* = 20)

	Median or number (IQR or %)
Age, *years*	66 (58–74)
Gender (women/men)	11/9 (55%/45%)
BMI, kg/m^2^	24 (20–26)
Clinical preoperative jaundice	10 (50%)
Preoperative biliary stenting	10 (50%)
ASA score I/II/III	1/13/6 (5%/65%/30%)
Preoperative diabetes	2 (10%)
Active smoker	7 (35%)
Ductal adenocarcinoma*	13 (65%)

*BMI*, body mass index; *ASA*, American Society of Anesthesiologists; *IQR*, interquartile range

Other etiologies were cholangiocarcinomas (*n* = 3), ampullary carcinomas (*n* = 2), and duodenal carcinomas (*n* = 2)

**Table 2:** Surgical, pathological, and postoperative details of the patient cohort (*n* = 20)

	Median or number (IQR or %)
Operation time, *min*	366 (318–386)
Portal vein resection	4 (20%)
Tumor size, *mm*	30 (24–47)
T stage 1/2/3/4	0/9/10/1 (0/45%/50%/5%)
N stage 0/1/2	4/6/10 (20%/30%/50%)
Grading 1/2/3	1/7/12 (5%/35%/60%)
Resection status R0/R1	11/9 (55%/45%)
Complications	13 (65%)
Major complications*	3 (15%)
Comprehensive complication index (CCI)	20.9 (8.7–27.9)
Delayed gastric emptying**	8 (38%)
Pancreatic fistula**	2 (10%)
Hemorrhage**	1 (5%)
Surgical-site infection°	2 (10%)
90-day readmission^#^	3 (15%)

*No postoperative mortality occurred during the first 90 postoperative days

**Based on the definitions of the international study group for pancreatic surgery (ISGPS) [13–15]

°Both infections were treated with antibiotics

^#^Two patients were readmitted for cholangitis that was treated with antibiotics and one patient was readmitted for abdominal pain necessitating intravenous analgesics due to early tumor recurrence

### Questionnaires inquiring about clinical exocrine pancreatic insufficiency

While 20 patients (100%) answered the preoperative questionnaires, 18 patients were available to answer the 1-year postoperative questionnaires (90%, 2 patients did not fill in the questionnaires). Table 3 summarizes the results of the pre- and postoperative questionnaires, assessing the presence of clinically relevant symptoms associated with exocrine pancreatic insufficiency. The only significant difference between preoperative and postoperative assessment was the frequency of constipation (less frequent 1 year after PD). All other items did not show any statistically significant difference. Of note, among the 17 patients who received adjuvant chemotherapy, only 4 of them were receiving or had been receiving chemotherapy at time or 6 months prior to the 1-year questionnaire filling.

**Table 3:** Results of the questionnaires preoperatively and 1 year after the operation. Answers were graded on a 5-point Likert scale from 1 to 5 (1 = always, 2 = most of the time, 3 = sometimes, 4 = rarely, 5 = never)*

	Preoperative (*n* = 20)	One-year postoperative (*n* = 18)	*p*-value
Abdominal discomfort	3 (3–4)	4 (3–4)	0.494
Bloating	4 (3–5)	4 (3–5)	0.501
Stomach heaviness after meals	3 (3–4)	4 (3–5)	0.092
Abdominal pain	4 (3–5)	4 (3–4)	0.723
Steatorrhea	4 (3–4)	3 (3–5)	0.988
Soft stools	3 (2–3)	2 (2–3)	0.502
Smelling stools	3 (2–4)	2 (2–5)	0.564
Diarrhea	3 (3–5)	4 (4–5)	0.136
Urgency	3 (3–5)	3 (2–4)	0.315
Constipation	4 (4–5)	5 (5–5)	**0.017**
Intestinal gaz	3 (2–3)	2 (2–3)	0.192
Loss of appetite	4 (3–5)	5 (3–5)	0.299
Eating with appetite and pleasure	2 (2–3)	1 (1–2)	0.053
Nausea	5 (4–5)	5 (4–5)	0.790
Weight loss*	3 (2–4)	2 (1–4)	0.267

Data are shown as median with interquartile range. Statistically significant values appear in bold. Questionnaires evaluated the different items within the last 6 months

*Regarding weight loss, the graduation was the following: 1 = no, 2 = <5 kg, 3 = between 5 and 10 kg, 4 = between 10 and 15 kg, 5 = >15 kg)

### Fecal elastase tests

Twelve patients (60%) had a preoperative fecal elastase test. It was not possible to obtain preoperative fecal elastase test in 8 patients due to logistic reasons. Median preoperative elastase level in the stools was 96 μg/g (IQR 15–196, *N* > 200 μg/g). Elastase tests realized 1 year after PD were performed in 14 patients (70%). Median time from PD to postoperative fecal elastase test was 13 months (IQR 12–15). Median postoperative elastase level in the stools was 15 μg/g (IQR 15–26). The median postoperative value was significantly lower than the median preoperative value (*p* = 0.042, Supplementary Fig. 2). Nine out of 14 patients (64%) had postoperative elastase level <15 μg/g, indicating severe insufficiency of the exocrine pancreas. In the two patients who developed postoperative pancreatic fistula, the 1-year elastase levels were <15 μg/g and 33 μg/g. If only patients with ductal adenocarcinoma are considered (*n* = 13), the median 1-year elastase level was 15 μg/g (IQR 15–16.25).

### Magnetic resonance imaging before and after intravenous secretin injection

Nineteen patients had a preoperative MRI with injection of gadolinium as part of the diagnosis measures and routinely performed tumor staging (95%). Sixteen patients (80%) had an MRI with i.v. secretin injection postoperatively, at 1 year after PD (2 patients refused and 2 were followed up at another hospital). Median time from PD to postoperative MRI was 13 months (IQR 13–14).

On 1-year MRI, median MPD size was not different before and after secretin injection for both investigator radiologists (radiologist 1: 4 mm, IQR 3–5 *vs*. 4 mm, IQR 3–5, *p* = 0.892 and radiologist 2: 3 mm, IQR 2–5 *vs*. 4 mm, IQR 3–6, *p* = 0.073). There were no transient MPD dilation (0/16), no differences before and after secretin injection in terms of MPD border regularity, MPD stenosis, side branches dilatation, MPD visibility, and MPD image improvement (*p* > 0.05). Both radiologists considered image quality slightly improved after i.v. secretin injection (radiologist 1: major improvement in 5 patients, slight improvement in 10 patients, and no improvement in 1 patient; radiologist 2: major improvement in 5 patients, slight improvement in 7 patients, and no improvement in 4 patients).

MRI results after secretin injection are summarized in Table 4. Fifteen patients had a patent pancreaticojejunostomy, while both radiologists considered that one patient had no permeability of the pancreaticojejunal anastomosis. Overall interobserver agreement kappa between the two radiologists was good (kappa coefficient: 0.72, 95% CI 0.67–0.77). Both patients who had postoperative pancreatic fistula had patent anastomosis on 1-year secretin MRI. Patients with ductal adenocarcinoma (*n* = 13) had similar patency results as the entire cohort on 1-year MRI (permeability rate: 12/13 = 92%).

**Table 4:** Magnetic resonance imaging findings after secretin injection (*n* = 16) as evaluated by each radiologist

	Radiologist 1	Radiologist 2
MPD size, *mm*	4 (3–5)	4 (3–6)
Regularity of the MPD borders		
Regular	14 (87%)	8 (50%)
Slightly irregular	2 (13%)	6 (37%)
Mainly irregular	0	2 (13%)
MPD focal stenosis	1 (6%)	0
Secondary duct dilation	7 (44%)	7 (44%)
Pancreaticojejunal anastomosis permeability	15 (94%)	15 (94%)
Jejunal filling 10 min after injection		
Two first jejunal loops or more	11 (69%)	13 (81%)
First and second jejunal loops	4 (25%)	2 (13%)
No secretion or limited to anastomosis	1 (6%)	1 (6%)
T1 parenchymal signal*		
Hypointense	8 (53%)	5 (33%)
Isointense	1 (7%)	8 (53%)
Hyperintense	6 (40%)	2 (14%)
Parenchymal atrophy (<15 mm**)	9 (56%)	8 (50%)
General image quality		
Poor	1 (6%)	0
Sufficient	3 (20%)	2 (12%)
Good	6 (37%)	10 (63%)
Very good	6 (37%)	4 (25%)

*MPD*, main pancreatic duct

Data are shown as median with interquartile range or number with percentage

*One patient had no T1 sequences

**Anteroposterior diameter of the pancreatic body

### Correlations between questionnaires, fecal elastase tests, and MRI

No significant correlation was observed between jejunal filling after secretin injection detected on 1-year MRI and 1-year fecal elastase dosage (rho: 0.16, *p* = 0.660) and between jejunal filling and steatorrhea reported on the questionnaire (rho: 0.235, *p* = 0.488). There were also no significant correlations between pancreatic atrophy as seen on 1-year MRI and 1-year fecal elastase dosage (rho: −0.42, *p* = 0.226) and between 1-year elastase dosage and steatorrhea reported on the questionnaire (rho: 0.239, *p* = 0.505).

## Discussion

This prospective cohort study assessing the 1-year outcomes of pancreaticojejunal anastomosis after PD suggests high patency rate of pancreatic anastomosis (94%) 1 year after PD observed at secretin MRI. The median fecal elastase test 1 year after PD, however, was significantly lower than preoperatively (15 *vs.* 96 μg/g, *p* = 0.042) and 9 patients had severe exocrine pancreatic insufficiency 1 year after PD.

The present study displayed that all patients but one (15/16, 94%) had a patent pancreatic anastomosis with jejunum filling as assessed at 10 min after secretin injection on 1-year MRI. This result was in contrast with the median value of the 1-year fecal elastase that was significantly decreased compared to preoperative value. These findings suggest that even if radiologically the pancreatic anastomosis is permeable, pancreatic exocrine insufficiency occurs. Therefore, postoperative pancreatic exocrine insufficiency would be explained by reduced secretion of the remnant pancreas or pancreatic atrophy, but not by obstructive problem. This pathophysiological mechanism was demonstrated in non-operated patients with exocrine pancreatic insufficiency and absence of MPD stenosis [19, 20]. A similar study evaluating secretin-stimulated MRI after PD described that permeability of pancreatic anastomosis was present in 70% of the patients (14/20) without specifying if the rest of the patients had no patency or if the images were not interpretable [8]. In addition, only one patient was diagnosed with MPD stenosis and underwent endoscopic treatment. Similarly, Hashimoto et al. found that only 2 out of 12 patients had strictures of the pancreaticojejunal anastomosis after PD [21]. These results corroborate the findings of the present study, where only one patient had radiological anastomotic stenosis.

One year after PD, median fecal elastase was 15 μg/g (IQR 15–26) compared to 96 μg/g preoperatively (IQR 15–196, *N* > 15 μg/g), indicating severe exocrine pancreatic insufficiency. Additionally, in the 1-year questionnaire, 50% of patients reported to have steatorrhea only occasionally. This discrepancy between postoperative fecal elastase test and symptoms (exocrine pancreatic insufficiency in 64% of the patients with a majority of them reporting occasional steatorrhea only) may be an argument in favor of postoperative pancreatic enzyme substitution. All patients received postoperative pancreatic enzyme substitution routinely (Creon) during the study period. A study by Lemaire et al. showed that all 17 PD patients with pancreaticogastrostomy had low fecal elastase within a median follow-up of 32 months, corroborating our results [22]. However, in a cross-sectional comparative study published in 2005, pancreaticojejunal anastomosis was found to induce less long-term exocrine pancreatic dysfunction (in particular less steatorrhea) compared to pancreaticogastric anastomosis [23].

Regarding 1-year pancreatic MRI, secretin injection did not change the morphology of the MPD in the present series, with good interobserver agreement. The advantage of secretin was a slight improvement of imaging quality on average. Few studies assessing the imaging improvement after secretin injection have been published in patients after PD. Monill et al. reported an improvement of image quality in 60% of the patients, but no difference for side branch detection, ductal narrowing, and ductal irregularities [8]. In another study assessing the role of secretin MRI after pancreaticojejunal anastomosis, the authors observed that secretin did not alter the visibility of the pancreatic duct itself but improved the assessment of the pancreaticojejunal anastomosis permeability [24].

This study represents a new comprehensive evaluation of pancreatic anastomosis 1 year after PD. It remains unclear if the anastomosis remains patent in the longer term, i.e., more than 1 year after PD, and if stenosis appears as late complication. A Japanese cohort study published in 1998 evaluated pancreaticogastric anastomoses by gastroscopy and pancreatography and confirmed patency in 4 out of 5 patients more than 9 years after PD [25].

Some limitations of the present study need to be mentioned. The study design was challenging, and our assessment included three different aspects, i.e., a functional, biological, and radiological component. These specificities complexified patient enrollment (high rate of refusal). Moreover, fecal elastase tests were difficult to obtain preoperatively because the tests were often performed the day before surgery at hospital admission. A stool sample was therefore not always available. The sample size of this study was rather modest, but data were collected prospectively, which improves the precision of the data collection and the data completeness. In addition, all included patients were fully investigated by three different methods (questionnaires, fecal elastase tests, and MRI), thus providing a whole functional, biological, and radiological evaluation of the pancreaticojejunal anastomosis at 1 year after PD.

## Conclusion

In conclusion, this study suggests that 1 year after PD, the pancreaticojejunal anastomosis remained patent in all but one patient and exocrine pancreatic secretion was impaired in 64%. Furthermore, as evaluated by the questionnaires, pancreatic enzyme replacement was effective in terms of symptomatology from the patient point of view.

### Supplementary information

ESM 1 (DOCX 15 kb)

ESM 2 (DOCX 1849 kb)

## Data Availability

All data generated or analyzed during this study are included in this article and its supplementary material files. Further enquiries can be directed to the corresponding authors.
