# Supplementary material for: Functional, biological, and radiological evaluation of the pancreaticojejunal anastomosis 1 year after pancreatoduodenectomy: a prospective study
Source: Langenbecks Arch Surg. 2023 Aug 22;408(1):326. doi: 10.1007/s00423-023-03040-x (PMC10444682; doi:10.1007/s00423-023-03040-x)
Supplement: Supplementary file 1 — (DOCX 15 kb) [file 423_2023_3040_MOESM1_ESM.docx]

**Questionnaire**

During the last six months, have you had:

1. abdominal discomfort?
2. always b) most of the time c) sometimes d) rarely e) never
3. abdominal bloating?
4. always b) most of the time c) sometimes d) rarely e) never
5. abdominal heaviness after meals?
6. always b) most of the time c) sometimes d) rarely e) never
7. abdominal pain?
8. always b) most of the time c) sometimes d) rarely e) never
9. greasy stools (floating in the toilets)?
10. always b) most of the time c) sometimes d) rarely e) never
11. soft stools?
12. always b) most of the time c) sometimes d) rarely e) never
13. stools with bad smell?
14. always b) most of the time c) sometimes d) rarely e) never
15. diarrhea (liquid and frequent stools)?
16. always b) most of the time c) sometimes d) rarely e) never
17. an urgent need to have a bowel movement?
18. always b) most of the time c) sometimes d) rarely e) never
19. constipation (less than 3 stools per week)?
20. always b) most of the time c) sometimes d) rarely e) never
21. gaz (flatulence)?
22. always b) most of the time c) sometimes d) rarely e) never
23. loss of appetite?
24. always b) most of the time c) sometimes d) rarely e) never
25. eaten with appetite and pleasure?
26. always b) most of the time c) sometimes d) rarely e) never
27. nausea?
28. always b) most of the time c) sometimes d) rarely e) never
29. weight loss?
30. no b) yes, <5 kg c) yes, >5 kg d) yes, >10 kg e) yes, >15 kg
