# Supplementary material for: Functional, biological, and radiological evaluation of the pancreaticojejunal anastomosis 1 year after pancreatoduodenectomy: a prospective study
Source: Langenbecks Arch Surg. 2023 Aug 22;408(1):326. doi: 10.1007/s00423-023-03040-x (PMC10444682; doi:10.1007/s00423-023-03040-x)

**Supplementary Figure 1.** Flow chart of the study

Patients undergoing PD during the study period (screened patients)

n=75

Patients excluded (n=53):

Refusal (n=25)

MRI contra-indication (n=11)

Not able to present the study to patients preoperatively (n=6)

Language barrier (n=6)

Chronic pancreatitis (n=5)

Patients included

n=22

Patients withdrawn (n=2):

PD not performed (n=1)

Pancreaticogastrostomy performed (n=1)

Patients included for final analysis

n= 20

Patients with preoperative MRI

n=19

Patients with preoperative fecal elastase test

n=12

Patients with preoperative questionnaires

n=20

Patients with postoperative secretin MRI

n=16

Patients with one-year fecal elastase test

n=14

Patients with one-year questionnaires

n=18

PD: pancreatoduodenectomy; MRI: magnetic resonance imaging.

**Supplementary Figure 2.** Box plot of preoperative and one year after pancreatoduodenectomy dosages of fecal elastase. Median preoperative elastase was significantly higher than median elastase one year after pancreatoduodenectomy (96 ug/g *vs.* 15 ug/g, p=0.042).


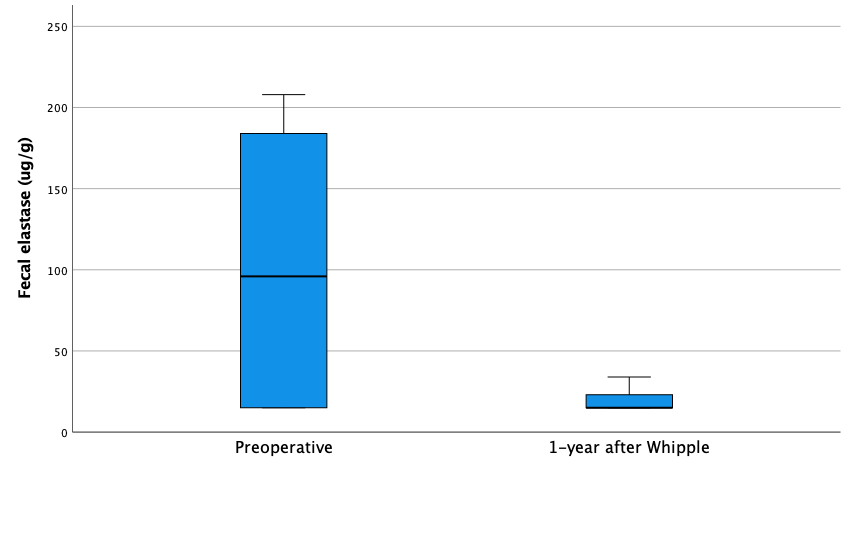

Supplement: Supplementary file 2 — (DOCX 1849 kb) [file 423_2023_3040_MOESM2_ESM.docx]
